# Environmental Influence on the Evolution of Morphological Complexity in Machines

**DOI:** 10.1371/journal.pcbi.1003399

**Published:** 2014-01-02

**Authors:** Joshua E. Auerbach, Josh C. Bongard

**Affiliations:** 1 Laboratory of Intelligent Systems, École Polytechnique Fédérale de Lausanne (EPFL), Lausanne, Switzerland; 2 Department of Computer Science, University of Vermont, Burlington, Vermont, United States of America; Indiana University, United States of America

## Abstract

Whether, when, how, and why increased complexity evolves in biological populations is a longstanding open question. In this work we combine a recently developed method for evolving virtual organisms with an information-theoretic metric of morphological complexity in order to investigate how the complexity of morphologies, which are evolved for locomotion, varies across different environments. We first demonstrate that selection for locomotion results in the evolution of organisms with morphologies that increase in complexity over evolutionary time beyond what would be expected due to random chance. This provides evidence that the increase in complexity observed is a result of a driven rather than a passive trend. In subsequent experiments we demonstrate that morphologies having greater complexity evolve in complex environments, when compared to a simple environment when a cost of complexity is imposed. This suggests that in some niches, evolution may act to complexify the body plans of organisms while in other niches selection favors simpler body plans.

## Author Summary

The evolution of complexity, a central issue of evolutionary theory since Darwin's time, remains a controversial topic. One particular question of interest is how the complexity of an organism's body plan (morphology) is influenced by the complexity of the environment in which it evolved. Ideally, it would be desirable to perform investigations on living organisms in which environmental complexity is under experimental control, but our ability to do so in a limited timespan and in a controlled manner is severely constrained. In lieu of such studies, here we employ computer simulations capable of evolving the body plans of virtual organisms to investigate this question *in silico*. By evolving virtual organisms for locomotion in a variety of environments, we are able to demonstrate that selecting for locomotion causes more complex morphologies to evolve than would be expected solely due to random chance. Moreover, if increased complexity incurs a cost (as it is thought to do in biology), then more complex environments tend to lead to the evolution of more complex body plans than those that evolve in a simpler environment. This result supports the idea that the morphological complexity of organisms is influenced by the complexity of the environments in which they evolve.

## Introduction

The “arrow of complexity” hypothesis [1] posits that the most complex products of open-ended evolutionary systems tend to increase in complexity over evolutionary time. Whether such a tendency exists is a long standing open question [2]–[6]. While it seems evident that more complex organisms exist today than at the advent of life, simple (single-celled) organisms continue to persist in large numbers, so it is clear that evolution does not guarantee complexity must increase. Moreover, loss of complexity has been observed in many species [7]–[9]. This begs the question: under what circumstances will complexity increase or decrease over evolutionary time? It is likely that particular environmental conditions are more likely to select for increased complexity than others, especially if this complexity comes at a cost.

As argued by proponents of embodied cognition, intelligent behavior emerges from the interplay between an organism's nervous system, morphology, and environment [10]–[14]. Therefore, if the ecological niche of a species remains constant and its body plan is evolutionarily constrained, then the neural system must adapt in order to succeed under this particular set of circumstances. This may be investigated experimentally through the use of evolving robots [15], [16] which stand in for biological organisms. For instance, it has been demonstrated [11], [17] that the complexity of an evolved neural system depends on the particular morphology it is controlling: in a given task environment certain morphologies can readily succeed with simple neural systems, while other morphologies require the discovery of more complex neural systems, or may prevent success altogether.

Another corollary of embodied cognition is that different environments will impose different selection pressures on the nervous systems and/or morphologies of organisms evolving in them. This can be studied by observing how organisms evolve in different environments. For instance, Passy [18] demonstrated that the morphological complexity of benthic colonial diatoms (measured as their fractal dimension) is significantly correlated with the variability of the environmental niches in which they are found. However, the biological evidence for a correlation between environmental and morphological complexity is sparse. This is in part because it is difficult to isolate systems where this may be studied effectively and to develop metrics that quantify morphological and environmental complexity. Ideally, it would be desirable to perform controlled investigations in which environmental complexity is under experimental control. Given enough time and resources it may be possible to carry out these investigations directly on living organisms. However, by performing experiments *in silico*, it is possible to do so with much greater speed and more precise control over experimental conditions. Specifically, by evolving virtual organisms [19] in physically realistic simulations, it is possible to faithfully model the relevant interactions between organisms and their environments.

Previously, the evolution of complexity has been investigated *in silico* using an alternative computational model [20]. In that work, populations of computer programs competed among themselves for the energy required to execute their instructions and gained energy by executing specific logic functions. With their system, Lenski et al. were able to demonstrate how complex functional features may evolve and how these features depend on the programs' environment. However, in that system the programs did not have bodies with which to physically interact with their environment. On the contrary, the evolutionary model employed here evolves embodied virtual organisms with evolutionarily determined body plans in physically realistic simulation environments. This provides a testbed for investigating how environment may influence the complexity of evolving physical morphologies.

Using *in silico* evolution to act on both the morphologies and nervous systems of simulated organisms or robots was first demonstrated by Sims [19], and has since been followed by a number of other studies (e.g. [21]–[32]). These studies employed a variety of experimental techniques, including different genetic encodings, morphological systems (such as branching structures or cellular aggregations), and evolutionary models. However, by constructing morphologies out of a relatively small number of geometric primitives, all of these studies were severely limited in the complexity of the morphologies which they could evolve, and therefore do not offer good test beds for investigating morphological complexity.

Recently, we introduced a new method for evolving virtual organisms that is capable of producing a greater diversity of morphologies than previous systems [33]. By using it to evolve organisms with restricted nervous systems in a variety of environments it was possible to demonstrate how such a system could be used for investigating the relationship between environmental and morphological complexity. Here, the results of [33] are refined and extended to demonstrate that selection for locomotion tends to induce selection pressures favoring more complex morphologies than would be expected solely due to random chance, and is therefore a driven rather than passive trend [3], [6], [34]. In subsequent experiments we employ a multi-objective selection mechanism to select for simplicity in addition to behavioral competency. This selection mechanism filters out morphological complexity that arises due to biases in the underlying evolutionary model or because of genetic drift, and only allows for complexity that confers a selective advantage on the simulated organism. Moreover, this selection mechanism acts to impose a cost on complexity as is thought to occur in biological organisms [35], [36]. Under this regime complex environments tend to induce selection for greater morphological complexity when compared to a simpler environment. This result supports the hypothesis that the environment plays an active role in determining morphological complexity.

In this work organisms are evolved in a variety of simulated environments in order to better understand the role of the environment in shaping morphological complexity. While inspired by the above mentioned studies in which the morphologies and controllers of virtual organisms were also evolved [19], [21]–[32], the system presented here has several advantages which make it better suited for studying the evolution of morphological complexity.

The first advantage relates to the task environments within which organisms evolve. The majority of the studies mentioned above were restricted to evolving for locomotion over flat terrain. While investigating this task has yielded interesting results, it suffers from its simplicity: simple morphologies composed of just a few cuboids or spheres are all that are needed to be successful. Even when more challenging task environments have been explored (e.g. those investigated in [37]), they employed morphologies composed of a small collection of cuboids and therefore the maximum complexity of their evolved morphologies was severely limited. In the current work, a variety of task environments with interesting properties are investigated, and morphologies with greater geometric detail are used, so it is possible to study the evolution of morphological complexity.

**Figure 1:**
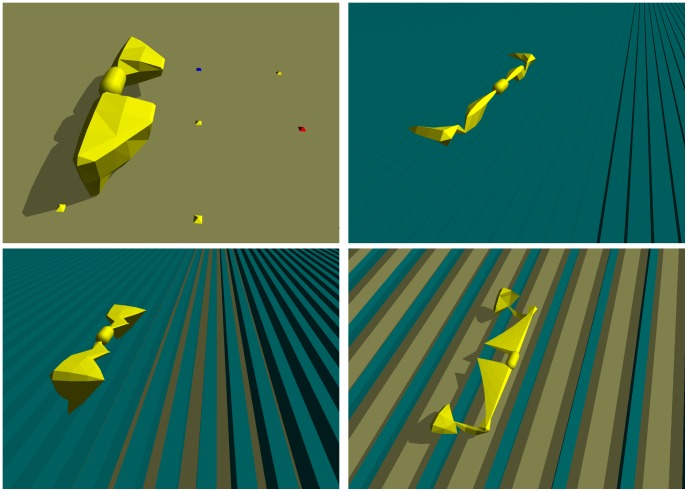
Evolved organisms and their environments. The control environment (top left) and three icy environments are shown with organisms that evolved to successfully move in each. The control environment only contains a high friction ground surface, while the icy environments feature low friction “blocks of ice” (blue) on top of the ground. Videos of these organisms are included in the Supplementary Material (Videos S1, S2, S3, S4, S5, S6, S7, S8, S9, S10, S11, S12, S13, S14, S15).

**Figure 2:**
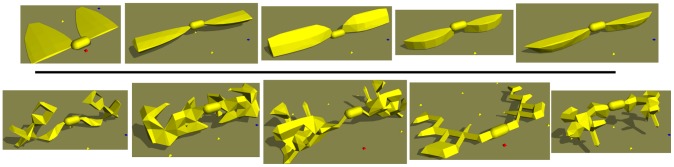
Simple and complex morphologies. The five morphologies with smallest (*top*, 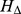 values from left to right: 0.66, 0.76, 0.82, 0.88, 0.88) and largest (*bottom*, 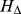 values from left to right: 3.74, 3.77, 3.80, 3.84, 3.84) values of 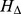 (see Methods for details) across all best of trial individuals from all environments (icy and control). The morphologies with high 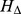 values are visually more complex than those with small 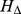 values.

Another advantage of the current system is the way in which the genetic material that the evolutionary model acts on is encoded. As has been demonstrated in the past [25], [26], genetic encodings that simulate development to some extent offer demonstrable benefits over those that do not. This is because such encodings tend to produce regularities and symmetries in the phenotype; such patterns in nature are the inevitable result of biological development, which biases the kinds of phenotypes that biological evolution may act on [38]. For this reason, here we employ a particular form of genetic encoding that produces three-dimensional shapes with regular patterns (see Methods for more details) [39]. Each genome generated from this encoding generates a triangular mesh (trimesh) that forms the body plan of the virtual organism. Trimeshes allow evolution to craft morphologies with greater geometric detail compared to other systems in which evolution composes a small number of simple three-dimensional shapes together [19], [21]–[32] (see Figs. 1 and 2 for examples of morphologies evolved with the current system). Finally, populations of these genetic encodings are evolved with a commonly-used evolutionary model which has been demonstrated to be more evolvable than other evolutionary models [40].

The behavior of each virtual organism is simulated in a three-dimensional, physically-realistic virtual environment in order to assess its fitness. Because of the organisms' triangular mesh body plans and the complex environments in which they are evolved, evaluating the fitness of each organism requires considerable time. Moreover, many evolutionary trials were conducted in each of several environments to allow for meaningful statistical analysis. For these reasons all of the experiments were carried out on a 7.1 teraflop supercomputing cluster and required a total of over 100 CPU-years of distributed compute time.

## Results/Discussion

In order to study the relationship between the morphological complexity of the virtual organisms and the task environments within which they evolve, evolutionary trials are conducted in each of 50 different environments. The first environment in which organisms are evolved is composed only of a uniform, flat, high friction ground surface (refer to Fig. 1a). The organisms evolved in this simple environment are considered control cases to compare against organisms evolved in other environments. Subsequent environments are more complex: they all consist of an infinite series of low friction rectangular solids over which an organism must locomote (see below for a characterization of this complexity). These “ice blocks” are constructed such that it is impossible for an organism to gain purchase by moving over their upper surfaces, but must instead reach into the gaps between the blocks to propel themselves forward in some fashion. This requires the evolution of morphologies with appropriate physical forms. Fig. 1 shows a sampling of these environments and virtual organisms that evolved within them.

The icy environments vary according to two parameters: the height of the blocks and the spacing between them. Each of these parameters varies from 0.025 meters to 1.6 meters exponentially for a total of 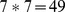 different environments. These two parameters and the their exponential scaling are employed in order to produce a variety of qualitatively different environments that roughly approximate natural surfaces, but yet are also amenable to analysis and efficient simulation. There are certainly many ways in which the environments could be created to more closely approximate natural terrain, and there are many other factors which could influence the complexity of an environment, however the parameterization employed here provides a set of environments within which it is largely possible to evolve organisms capable of successful locomotion with the bare minimum of neural complexity. This allows for isolating the influence of environment on morphological complexity, which is the property of interest in this study (see Conclusions for further discussion).

**Figure 3:**
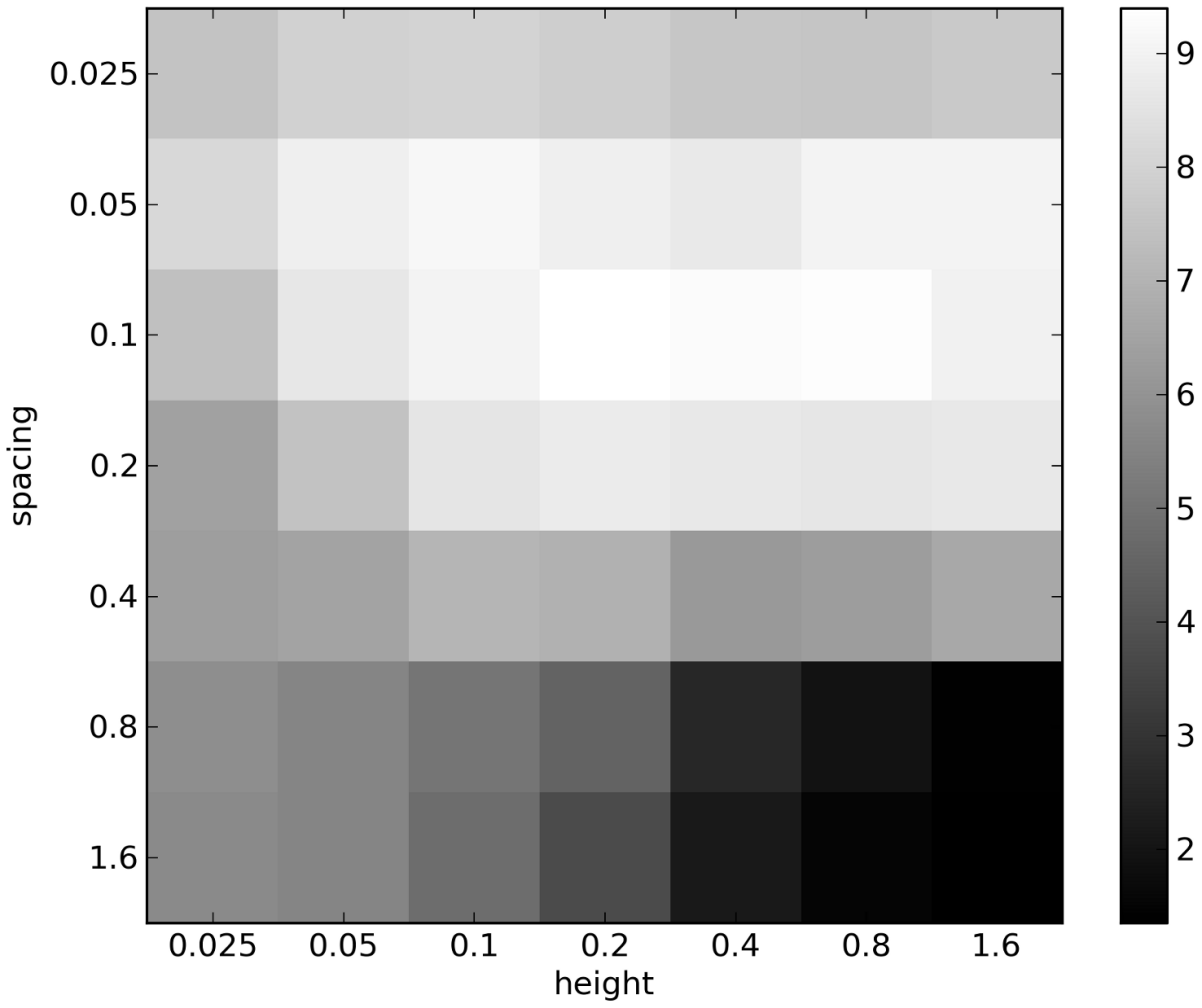
Mean distance achieved in each environment. This plot shows the mean distance achieved by the final generation champion taken across the 100 independent trials of CPPN-NEAT in each of the 49 icy environments. For comparison, the mean distance achieved across all 4900 independent trials in the control environment is 7.32 meters.

For each icy environment, 100 evolutionary trials are conducted in that environment and a corresponding 100 evolutionary trials are conducted in the control environment (for a total of 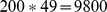 evolutionary trials; see Methods for details). Fig. 3 reports the mean distance that the best individuals from each trial traveled (computed across the 100 independent trials) in each icy environment. This figure demonstrates that there is a clear relationship between the environmental parameters and the difficulty of the task. Specifically, moving to the lower right in Fig. 3, where both the spacing and the height of blocks are large, the task becomes increasingly difficult: the organisms all become trapped in the gaps between blocks. Keeping the spacing constant and decreasing the block height (moving left in Fig. 3) gradually eases the task: the organisms are able to navigate over these smaller blocks and displace themselves at least several body lengths. Once the height has been reduced to 0.025 meters the blocks are so short that the environment becomes very similar to flat ground, and in fact distances achieved by organisms in the lower left environments are not significantly different from those of the control environment.

As the spacing between the blocks is reduced (moving upward in Fig. 3) the organisms are no longer able to behave as they would on flat ground, but instead must find ways to move along the tops of the blocks while finding a means of gaining purchase by reaching into the gaps. The height of the blocks loses importance in this part of the parameter space but still has an effect (though opposite to when the spacing is large). Here the general pattern is for taller blocks to make the task easier, because taller blocks provide more voluminous gaps which more easily support a variety of ways to gain purchase. Finally in the top row of Fig. 3, when the spacing is smallest, block height ceases to have much of an impact because however narrow an organism's appendages are they can only reach a short distance into the gaps.

For a better understanding of how the evolved organisms behave in each of these environments it is helpful to observe their behavior. For this purpose, sample videos of evolved organisms are available in the Supplementary Material (Videos S1, S2, S3, S4, S5, S6, S7, S8, S9, S10, S11, S12, S13, S14, S15).

### Quantifying Complexity

It is clear that different environments in this parameterization present the evolutionary system with varying degrees of difficulty, but the question now becomes: how does environment influence the evolution of morphological complexity? There are many approaches to quantify the complexity of an evolved morphology. Commonly, the variability of part types such as the number of cell types [41] has been used to measure the morphological complexity of biological organisms. But, the parts under consideration may vary in scale from organelles [42] to limbs [43], and it is unclear what should be considered a part in the current work. More geometric measures describing how space-filling a morphology is could also be employed (see Text S1 and Figure S2). Alternatively, a morphology's surface area to volume ratio could be measured, or its concavity could be computed (e.g. by taking the ratio of a morphology's volume to that of its convex hull). However each of these measures may be deceived by relatively simple body shapes, such as those that are very flat or contain large, simple concavities (e.g. a ‘C’ shape).

Instead, it is useful to think about the complexity of a body plan in information theoretic terms. One commonly used measure of complexity is Shannon's Entropy [44], which measures the uncertainty of a random variable. Recent work [45], [46] has demonstrated how Shannon Entropy can be applied to measure the complexity of a 3D object by considering the curvature of the object as a random variable. This means that in order to have higher complexity it is necessary to have more angles (regions of non-zero curvature) that can not simply be a repeating pattern, exactly what humans would think of as more complex shapes. And in fact, quantifying the complexity of 3D objects in this way has been shown to strongly correlate with human observers' notions of complexity [46].

In this work, the complexity of an organism's morphology is computed as the quantity 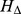 which is the morphology's entropy of curvature or, in terminology which may be more familiar to biologists, it is the Shannon diversity [47] of the curvature on the organism's exterior (see Methods for details). Does 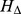 capture the complexity of evolved morphologies? To answer this question, 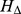 is calculated for all 9800 best-of-trial virtual organisms from all environments (icy and control). Out of those 9800, the five morphologies with the smallest 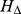 value and the five morphologies with the largest 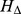 value are selected. Images of these morphologies are shown in Fig. 2. Looking at these two sets of morphologies, those with high 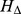 values appear more complex than those with low 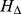 values. In light of this observation and the previous work in this area it is concluded that 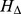 successfully captures morphological complexity.

Similarly, the concept of entropy may also be applied to characterizing the complexity of an environment. In the current formulation, environments are differentiated by variability in surface friction and terrain elevation. In the flat ground environment both the height of the terrain and the surface friction are uniform throughout, thus conveying zero entropy. On the other hand, in all of the icy environments there is variability in both of these properties. The surface friction is low on the ice blocks, but high on the ground between them. Likewise, the terrain is one height on the blocks and another in the intervening space. Therefore each of the icy environments has non-zero entropies of friction and elevation and so is considered to be more complex than flat ground. However, since each icy environment consists of a uniform series of ice blocks, the relative complexity between these environments is not considered.

### Changes in Complexity over Evolutionary Time

**Figure 4:**
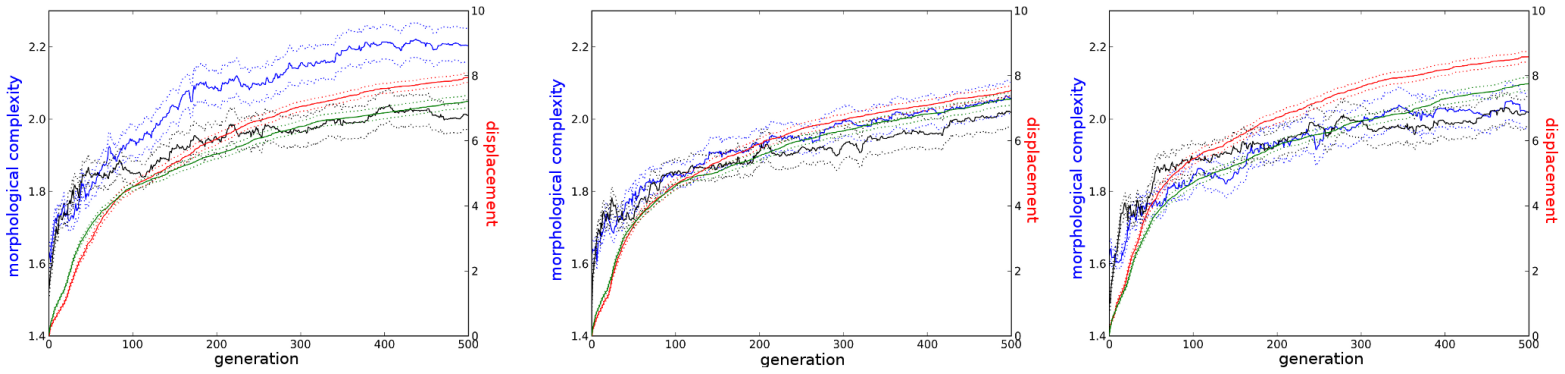
Sample complexities and fitnesses over evolutionary time. This plot depicts morphological complexity (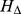) and fitness (displacement) over evolutionary time for three sample icy environments (**left**: spacing 0.025, height 0.05, **middle**: spacing 0.025, height 0.8, **right**: spacing 0.2, height 0.8) along with their corresponding set of trials from the control environment. For the icy environments morphological complexity is plotted in blue and displacement is plotted in red. For the corresponding trials in the control environment morphological complexity is plotted in black and displacement is plotted in green. Solid lines denote means (taken across all best-of-generation individuals from all trials in the set) and dotted lines denote one unit of standard error.

Armed with these measures, it is now possible to characterize how different environments influence the morphological complexity of evolving organisms. In order to understand the evolutionary pressures which lead to virtual organisms that are more or less morphologically complex, it is interesting to consider how morphological complexity varies over evolutionary time in different environments, and how these changes correspond to variations in fitness. Towards that goal, Fig. 4 depicts the mean morphological complexity and mean displacement of the current best individual over evolutionary time for each of several icy environments along with a corresponding set of control trials. Here it can be seen that morphological complexity tends to increase over time along with fitness. This means that in these environments selection for locomotion corresponds to an increase in complexity.

However, it is unclear whether this increase of complexity is the result of a passive or a driven trend [3], [6], [34]. Passive trends may result from envelope expansion without any directional bias. For example, if there is a minimum level of complexity necessary for success, but no upper bound, then both the mean and the maximum complexity of the population will increase over time simply due to random variation (what Stephen Jay Gould famously referred to as a “drunkard's walk” [9]). On the other hand, driven trends exhibit a consistent, directional bias. This corresponds to active selection for greater complexity. In this case not only will there be an increase in mean and maximum complexity, but the minimum level of complexity will increase over evolutionary time as well.

### Neutral Shadow Model

**Figure 5:**
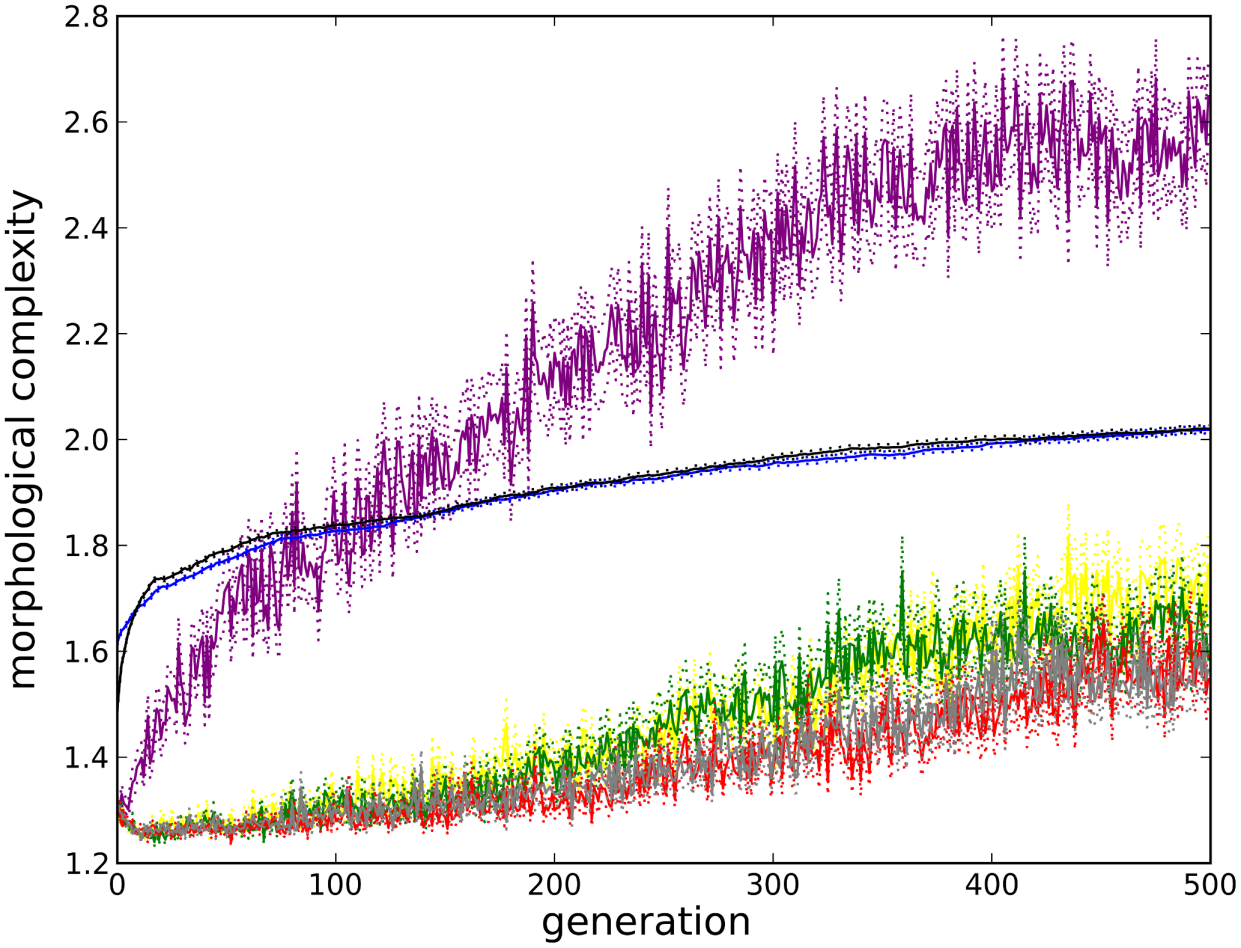
Complexity over evolutionary time versus neutral shadows. This plot compares morphological complexity (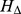) over evolutionary time for all single-objective experiments in the control environment (black) and all icy environments (blue) along with several neutral shadow models. Solid lines denote means (taken across all best-of-generation individuals from all trials in that environment) and dotted lines denote one unit of standard error. The purple line depicts the naïve shadow model: completely random selection except for a preference for valid morphologies. The remaining lines depict the alternate shadow models with reproduction depths matched to the two real evolutionary experiments (see Text S1 for details). Yellow = shadow model *a* matched to the control environment, green = shadow model *a* matched to an icy environment, red = shadow model *b* matched to the control environment, and gray = shadow model *b* matched to an icy environment.

When looking only at how morphological complexity varies over evolutionary time it is unclear what change in complexity is due to selection pressure from the environment and what change is due to biases towards increasing complexity within the evolutionary model itself and/or the general tendency of evolutionary systems to produce increasing complexity in the absence of selection [48]. In order to separate the influence of these factors it is useful to compare the evolving populations to a neutral shadow model [49], [50]. For a generational evolutionary model, such as that employed here, a neutral shadow of a given experiment is equivalent to re-running the evolutionary model with the same parameters but with random selection. Fig. 5 shows how the morphological complexity of organisms evolved in flat ground (black), as well as all icy environments (blue), changes over evolutionary time compared to those evolved in 100 independent trials using random selection (purple) in which the only preference is for genomes that produce valid morphologies (so that there exists a morphology for which complexity can be calculated; see Methods). It is known that the evolutionary system employed here [40] has an inherent bias to increase genotypic complexity over evolutionary time. The increasing purple curve in Fig. 5 indicates that there exists a bias to produce more complex morphologies over time as well. In fact, random selection alone produces morphologies that are more complex than those selected in any of the environments investigated. However, this comparison is not entirely fair. At any given generation, individuals in the random selection experiments will be the end product of many more reproduction (mutation and crossover) events than the corresponding individuals evolved for displacement, because under random selection it is unlikely that any individual will persist in the population for very long. Therefore, individuals in the random selection experiments will have had many more opportunities to increase the complexity of their genomes and hence the complexity of their morphologies.

In order to correct for this discrepancy in the number of reproduction events, alternative shadow models are employed. Specifically, neutral shadow models of both the flat ground experiments and a representative icy environment (spacing 0.025, height 0.8) are created, which control for the number of reproduction events leading to the individuals in the current population. In each of the 100 independent trials evolving for locomotion in both of these environments, a record of every reproduction event is kept, and alternative shadow models are created for each trial such that they maintain the same rate of reproduction. These shadow models are detailed in Text S1.

All model alternatives have similar complexity curves (see yellow, green, red and gray lines in Fig. 5) indicating that this shadow formulation is robust to whichever alternative is employed. Qualitatively they both show a much slower increase in morphological complexity (especially early on in evolution) compared to the experiments selecting for displacement, and so contrary to the naïve shadow model, both flat ground and icy environments select for increased morphological complexity beyond what would be expected in a neutral model. This implies that greater morphological complexity is being actively selected for in these environments: there is a driven trend towards increased morphological complexity.

### Multi-objective Selection

While the results reported so far support the hypothesis that there is a driven trend for increased morphological complexity in all environments, they do not differentiate between the complexities of organisms evolved based on which environment they are evolved in. Specifically, Fig. 5 depicts similar levels of complexity evolving in icy environments as compared to the flat, high friction environment under this regime. In fact, when the morphological complexities of organisms evolved in each of the 49 icy environments are compared with independent sets of trials conducted in the control environment (see Figs. 4 and S1) they do not reflect a consistent relationship between environment and evolved morphological complexity. It is hypothesized that without a cost to becoming more complex the driven trend towards increased morphological complexity will dominate in all of the investigated environments.

On the other hand, it is hypothesized that when complexity does come at a cost–as is thought to occur in biological organisms [35], [36] –there will be greater pressure towards increased morphological complexity in more complex environments. In an an attempt to test this hypothesis, a second set of experiments is conducted which uses Pareto based multi-objective selection [51], [52] to evolve organisms that can locomote in their given task environment and are as simple as possible, therefore imposing a cost on complexity.

As was done for the single-objective experiments, 100 independent trials of a multi-objective model are run in each of the 49 icy environments along with a corresponding 49 independent sets of 100 trials apiece in the high friction, flat ground control environment. By selecting for both maximal displacement and minimal morphological complexity these experiments should evolve organisms that are no more complex than necessary to succeed in their task environment. If indeed more complex environments induce greater selection pressure favoring morphological complexity than simple environments when morphological complexity comes at a cost, then these differences should be observable under this regime.

**Figure 6:**
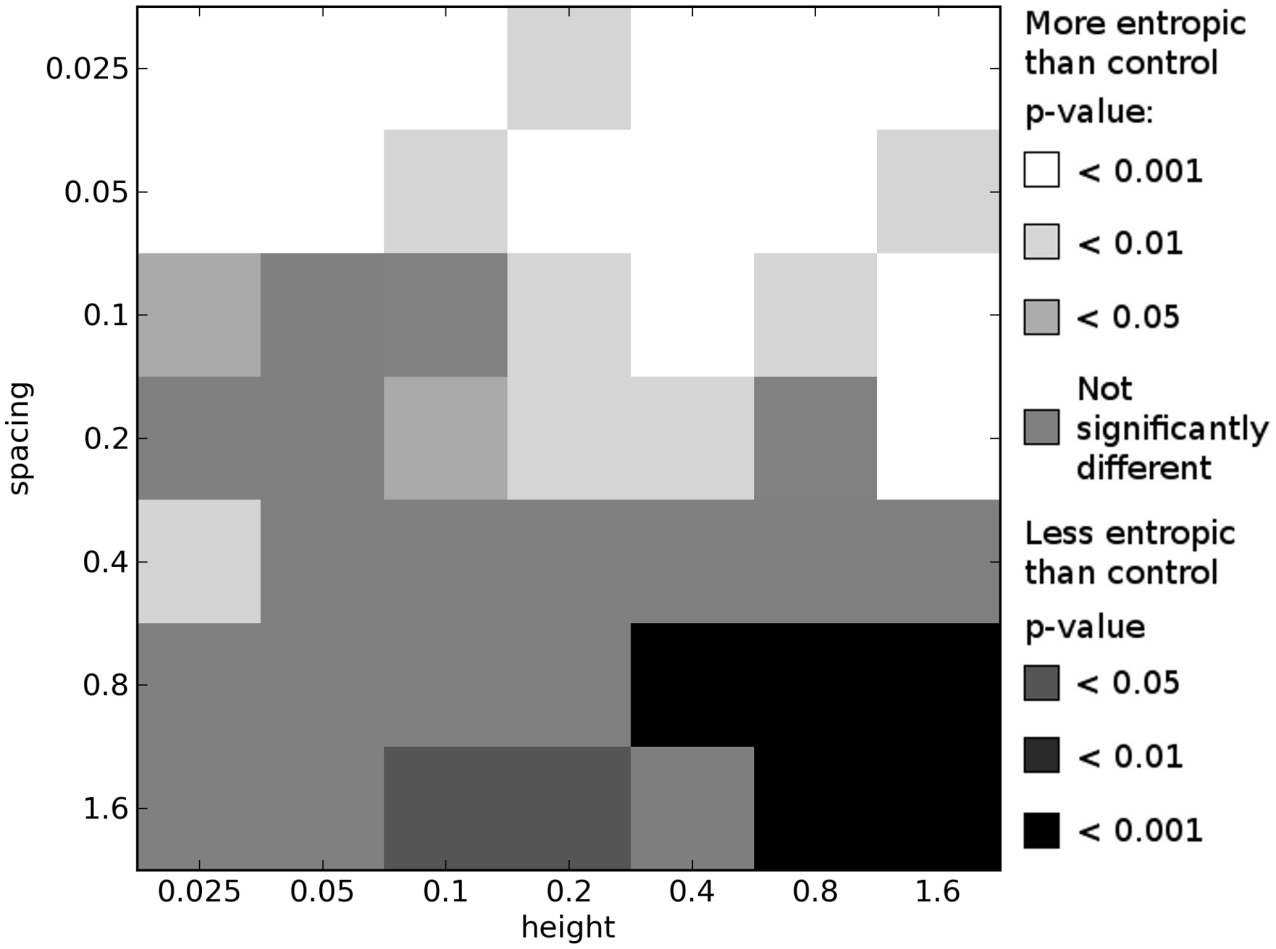
Differences in morphological complexity between icy and control environments: multi-objective means. This plot compares the ways in which the complexity of morphologies from icy environments differ from the complexity of morphologies evolved in the control environment under multi-objective selection. This plot is created from the multi-objective results by comparing the mean 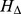 values across each trial's final Pareto front in each icy environment to the mean 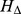 values across each trial's final Pareto front in a corresponding set of trials in the control environment. See Results/Discussion for details. All *p*-values calculated using the Mann-Whitney U test.

**Figure 7:**
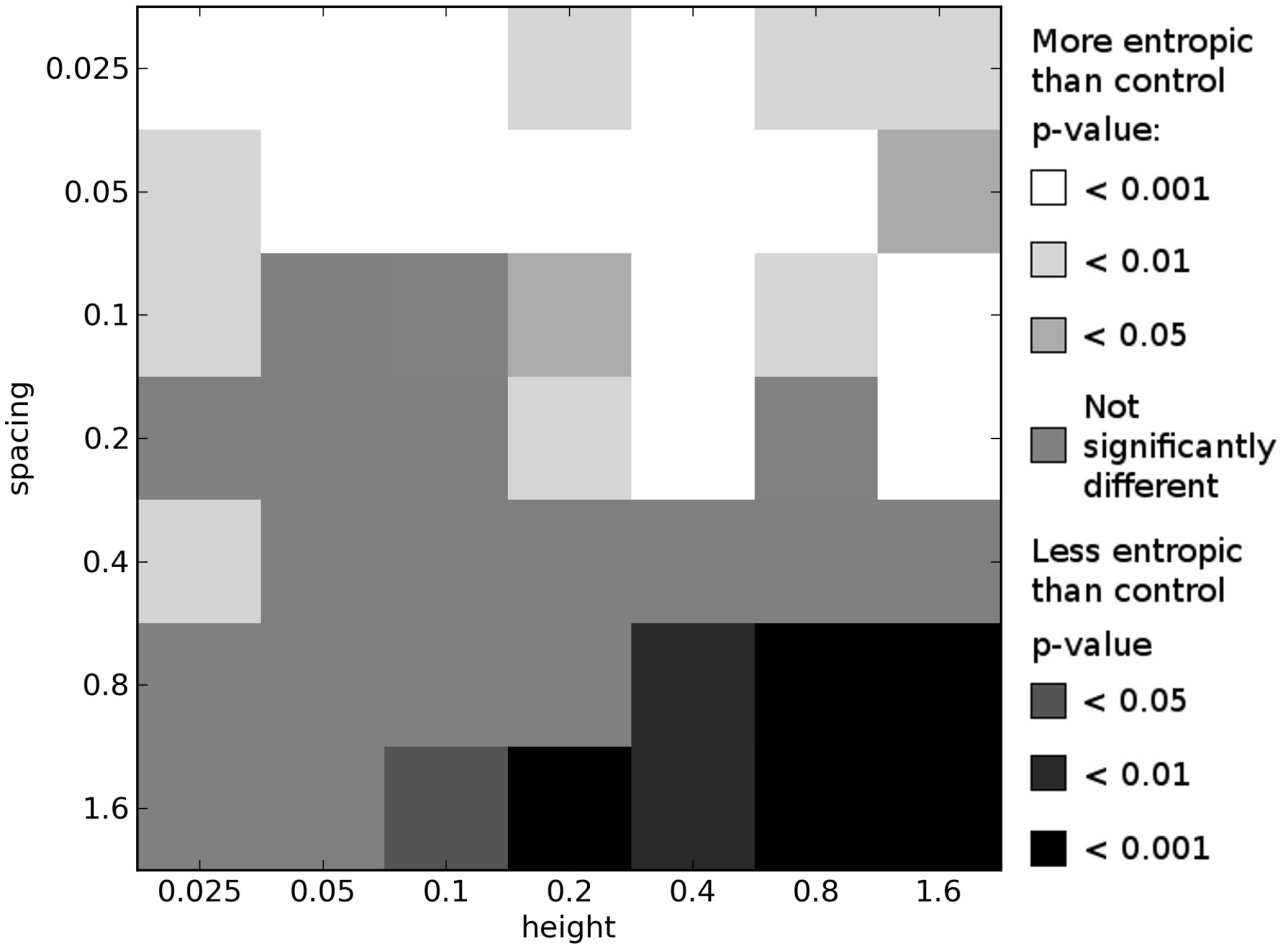
Differences in morphological complexity between icy and control environments: multi-objective medians. This plot compares the ways in which the complexity of morphologies from icy environments differ from the complexity of morphologies evolved in the control environment under multi-objective selection. This plot is created from the multi-objective results by comparing the 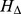 values of the median individual from each trial's final Pareto front in each icy environment to the 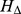 value of the median individual from each trial's final Pareto front in a corresponding set of trials in the control environment. See Results/Discussion for details. All *p*-values calculated using the Mann-Whitney U test.

**Figure 8:**
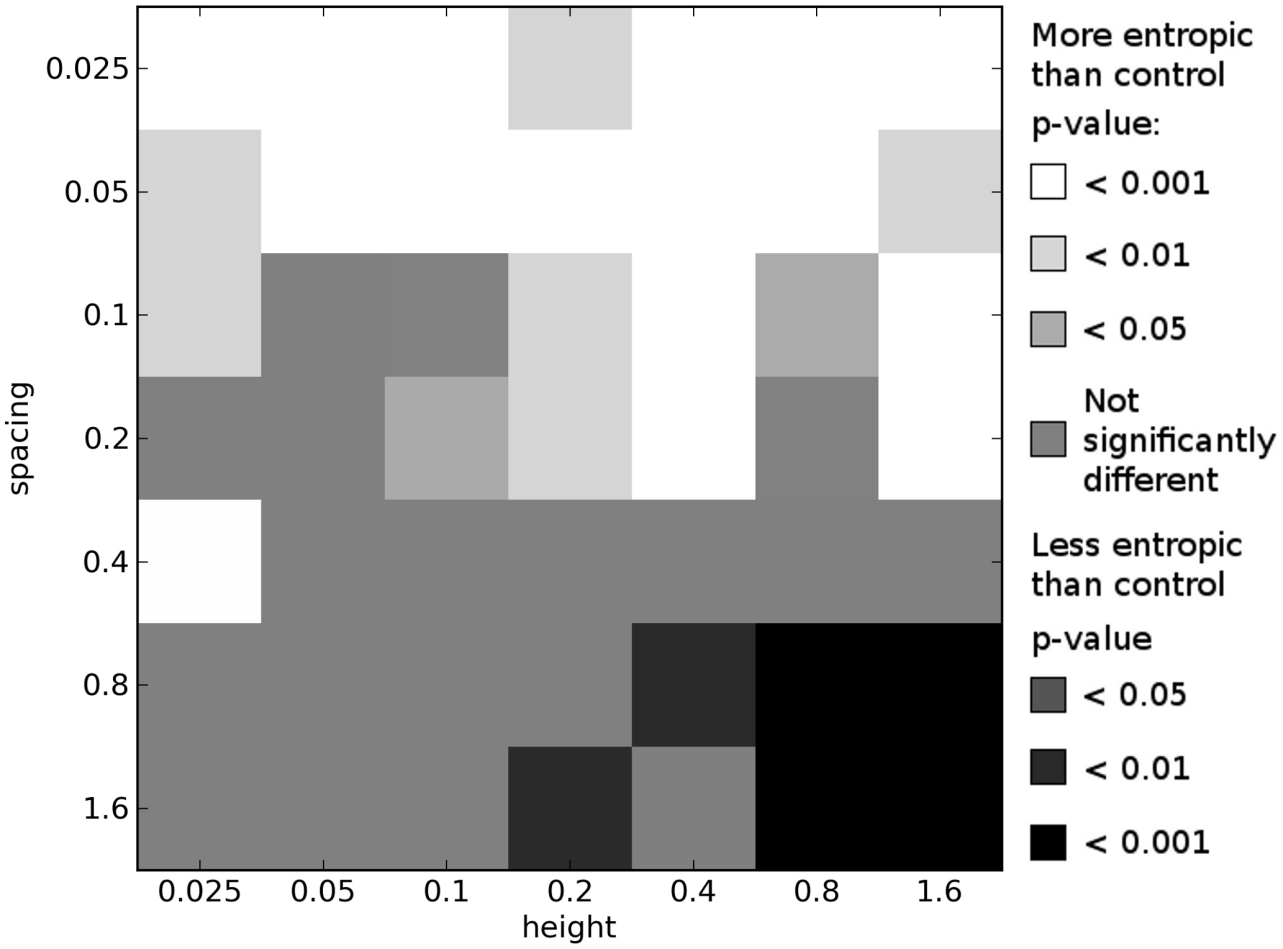
Differences in morphological complexity between icy and control environments: multi-objective means of center halves. This plot compares the ways in which the complexity of morphologies from icy environments differ from the complexity of morphologies evolved in the control environment under multi-objective selection. This plot is created from the multi-objective results by comparing the mean 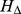 values across the center half of each trial's final Pareto front in each icy environment to the mean 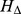 values across the center half of each trial's final Pareto front in a corresponding set of trials in the control environment. See Results/Discussion for details. All *p*-values calculated using the Mann-Whitney U test.

Comparing the results of these multi-objective experiments, we indeed see that more complex environments tend to select for organisms with greater morphological complexity when compared with organisms evolved in the simple, control environment. Figs. 6–8 show how the morphological complexities of organisms evolved in each of the icy environments under multi-objective selection differs from that of organisms evolved in a corresponding set of trials from the control environment. Since selecting a single representative individual from each trial is not as straightforward as in the single-objective case (see Methods), several different techniques are employed to compare the results of these experiments.

**Figure 9:**
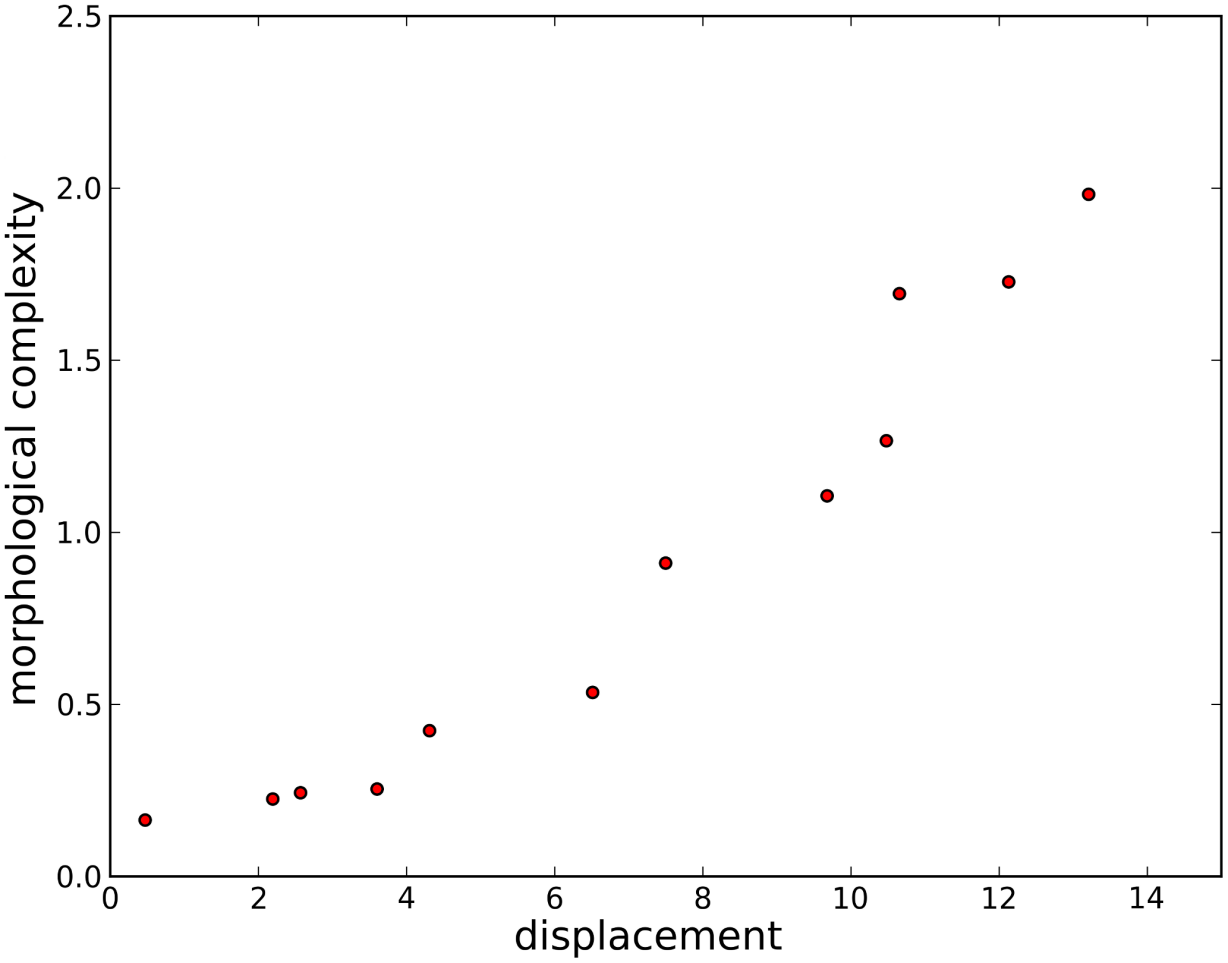
A sample Pareto front. This plot depicts the set of non-dominated individuals at the end of an evolutionary trial attempting to maximize displacement and minimize complexity. The points on the far left represent organisms that are very simple, but do not move far. The points on the far right represent organisms that are more complex, but also are able to move further.

First, for the final Pareto front of each trial in a given environment, the mean morphological complexity is taken. These means (100 from each environment) are compared to the mean morphological complexity in the final Pareto front of each trial from a corresponding set of trials from the control environment. This comparison is depicted in Fig. 6. Fig. 7 presents the same comparison except that it considers the organism with median performance on each Pareto front: the organism with equal number of individuals on the front that displace less and more than it (e.g. the most central point in Fig. 9). Lastly, Fig. 8 shows the same comparison except that it considers the mean complexity of those organisms in the middle half of their respective Pareto fronts. That is, the top quarter of the most complex morphologies (rightmost three points in Fig. 9) and the bottom quarter of most simple morphologies (leftmost three points in Fig. 9) in each front are ignored, and the means are taken across the remaining organisms in each front (which should reduce the influence of any outliers).

While some differences can be observed across these plots, the general pattern is largely consistent (and therefore not an artifact of the particular comparison employed): imposing a cost on complexity results in a multitude of icy environments where significantly more complex morphologies evolve compared to the control environment, and many of these differences are observed at the highest significance level (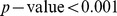). This corroborates the hypothesis that the more complex environments induce selection pressure for increased morphological complexity beyond what would evolve in a simpler environment when morphological complexity comes at a cost.

In the lower right of Figs. 6–8, where the environments become too difficult to succeed in (because the organisms get trapped in the large gaps; see Fig. 3), multi-objective selection actually results in the evolution of morphologies that are significantly less complex than those that evolve to locomote on flat ground. The reason for this is that when it is not possible to evolve for greater displacement, the majority of selection bears down on the simplicity objective, and therefore simpler morphologies evolve in these environments under multi-objective selection.

### Conclusions

This paper has presented a new method for evolving not only the neural systems but also the body plans of virtual organisms. This system differs from previous work by evolving populations of genetic encodings that produce complex morphologies instantiated in virtual environments as triangular meshes. This methodology opens up the possibility of investigating previously unexplored relationships between evolving organisms and their environments in a systematic manner.

Here, this system was used to investigate how different environments induce differing selection pressures on morphological complexity. By evolving virtual organisms in a number of different task environments and analyzing how an information theoretic measure of morphological complexity varies over evolutionary time, it was demonstrated that not only do all investigated environments actively induce selection pressure favoring greater complexity above and beyond what would be expected in the absence of selection, but that more complex environments in fact induce selection for more complex morphologies then simple environments when a cost is imposed on morphological complexity. Since it is often thought that complexity does incur a cost in biological organisms [35], [36], the differences observed between environments in this regime may be more representative of the selection pressures present in biological systems.

These results have illustrated how the environment may influence the complexity of evolving morphologies. Based on the results presented here it is possible that a similar evolutionary dynamic has been partially responsible for the “arrows of complexity” observed among biological organisms. As organisms have come to occupy more complex niches it is likely that these niches have actively selected for increased morphological complexity. Additionally, it should be possible to leverage this property for evolving more complex artifacts with evolutionary computation systems. However, it is not likely that increased environmental complexity will select for increased morphological complexity in every case where such complexity incurs a cost. While this work has demonstrated that such a relationship can exist, future work is needed to clarify this relationship across different environments, tasks, organisms, evolutionary models, and neural systems.

A number of simplifications were made here which it may be desirable to relax in future work. By constraining the number of morphological components and using very simple neural architectures it was possible to largely bracket the question of neural complexity and focus on one particular aspect of morphological complexity. However, it may be desirable to investigate how many different forms of complexity evolve as a function of environment. For instance, in a recent study [53] we demonstrated that another measure of complexity: “mechanical complexity”, decreased in the same environments that selected for greater morphological complexity. This result lends support to the notion that various forms of complexity may be inversely correlated as discussed in [54], and it also suggests that there is likely a trade-off between the various forms of complexity needed to succeed in a given environment, similar to the trade-off between morphological and neural complexity [11], [17].

To investigate these ideas further it will be interesting to allow for more complex neural architectures, more complex sensorimotor systems, and a greater diversity of materials (including ‘soft’ materials [55]) to study how environments may influence the evolution of sensorial, nuerological, motoric, material, mechanical, and morphological complexity of these various systems. By extending the information theoretic ideas used here for quantifying morphological complexity it is hoped that a ‘common currency of bits’ may be used to investigate these complexity trade-offs in a systematic manner.

## Methods

### CPPNs

The morphologies evolved in this work are encoded by Compositional Pattern Producing Networks (CPPNs) [39]. CPPNs are a form of artificial neural network (ANN) [56] which differ from traditional ANNs in several ways. While each internal node in a traditional ANN typically has the same activation function (such as a sigmoid or a step function), CPPN nodes can take on one of several activation functions from a predefined set. This function set often includes functions that are repetitive, such as 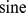 or 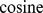, as well as symmetric functions, such as 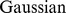, thus allowing for motifs seen in natural systems that arise as a result of development: symmetry, repetition, and repetition with variation. Additionally, CPPNs are often used as generative systems to produce other objects of interest, such as images [57], 3D structures [58], [59], robot morphologies [31], [32] or traditional ANNs themselves[60]–[64]. This is in contrast to the typical, direct application of ANNs as robot control architectures or classifiers.

CPPNs act as functions of geometry. Geometric coordinates meaningful to the object being represented are fed as inputs to the CPPN. These input values are passed through the various connections of the CPPN from node to node. Each node aggregates its inputs by taking a weighted sum of the values output by each upstream node (weights are specific to each connection) and outputs the result of applying a particular activation function (specific to that node) to this weighted sum. By passing the inputs through subsequent nodes the activation functions are composed to produce novel outputs while maintaining features of the different functions (hence the “compositional” aspect of CPPNs). Additionally, since these functions are chosen to have desirable properties present across a wide range of natural systems, as discussed above, CPPNs are capable of directly producing structures which in nature require a developmental process. For a more in-depth description of CPPNs, and further discussion of their ability to act as an abstraction of development, the reader is referred to [39].

### Evolutionary Model

In this study CPPNs are evolved via CPPN-NEAT [39]. CPPN-NEAT is an extension of the NeuroEvolution of Augmenting Topologies (NEAT) [40] method of neuro-evolution. NEAT is capable of evolving not only connection weights for existing network topologies, but also the network topologies themselves. Its operation is based on a few key ideas. First, the initial population is comprised of minimal networks (those without any internal or hidden nodes), which may then gradually increase in complexity over evolutionary time through structural mutations which add new nodes and links to the network. When a new node or link is created in this manner it is assigned a unique historical marking. These historical markings are inherited during reproduction and allow meaningful crossovers to occur without the use of expensive graph matching procedures. Additionally, these markings are used to divide the population into “species” of similar network topologies. Speciation promotes genotypic diversity and, because competition is primarily intraspecies, novel structural innovations are given time to mature before directly competing with individuals in other species.

CPPN-NEAT extends NEAT to evolve CPPNs. Effectively, this means that since nodes are no longer restricted to having sigmoid activation functions, each node contains an additional parameter which specifies its own activation function. When a new node is added to a network it is assigned a random function from a predefined set (the signed cosine, Gaussian and sigmoid functions are used in the experiments reported here). Additionally, the compatibility distance metric used for speciation is modified to incorporate the number of different activation functions between two networks. In all other respects, CPPN-NEAT behaves the same as NEAT.

NEAT and CPPN-NEAT have successfully evolved ANNs and CPPNs for a variety tasks [40], [57], [59], [61], [62], [65] which makes CPPN-NEAT a good option for evolving the CPPNs used in this study. Moreover, CPPN-NEAT's ability to systematically increase network complexity over evolutionary time as needed should lend itself well to studying how morphologies increase in complexity when evolving inside different environments. For a more thorough description of these algorithms, including additional details of the mechanisms discussed above, please refer to [39], [40].

### Building Morphologies from CPPNs

**Figure 10:**
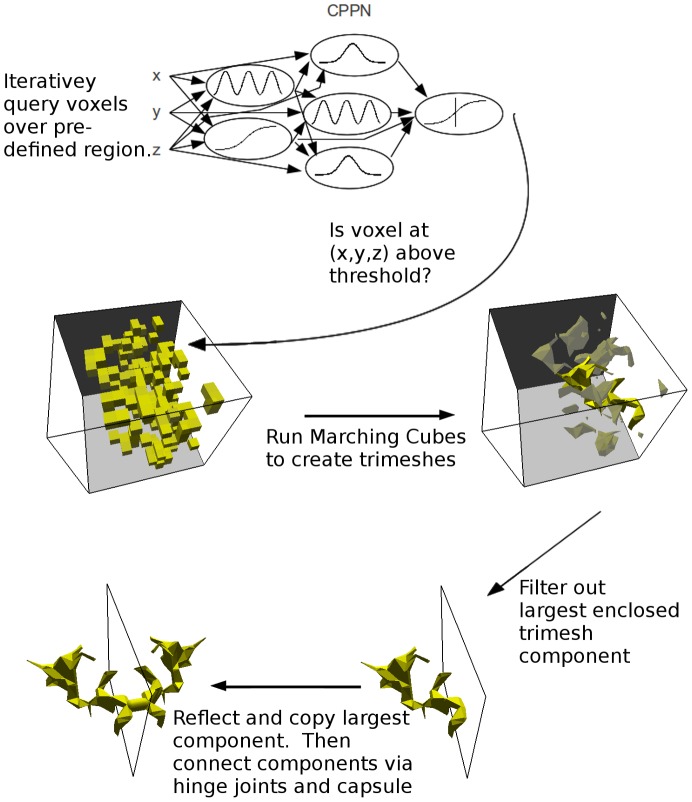
Constructing an organism from a CPPN genome. First a candidate CPPN is iteratively queried to find the output value at all voxel locations. These values are used as inputs to the Marching Cubes algorithm [66] which produces a set of enclosed trimesh components containing all voxels with output value above a given threshold. From the many enclosed components that may be produced, the largest (in terms of number of triangles) is selected. This trimesh component is then copied and reflected across the 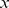-axis. The resulting components (the original and its mirror image) are then spread apart by 0.2 meters and a capsule is placed between them. Finally, the two trimesh components each connect to this capsule by means of a hinge joint which when combined form a universal joint capable of rotation through the organism's coronal and sagittal planes.

While previously [31], [32] evolving virtual organisms were constructed out of spherical components, the current study employs a voxel-based method to create morphological components out of triangular meshes (trimeshes) similar to what is done for the creation of 3D shapes in [59]. This process is illustrated in Fig. 10, and is explained in detail below.

First, A regular grid is placed over a region of 3D space which defines the presence of voxel locations. In the current work this region extends from 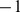 to 1 (inclusive) in each dimension and grid lines are placed at intervals of 0.2. This yields a total of 11 grid lines in each dimension for a total of 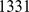 voxels. A candidate CPPN is iteratively queried with the 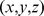 Cartesian coordinates at every voxel location except for the extrema in each direction. Querying a CPPN at a given location involves resetting all node values, and updating the CPPN for a fixed number of iterations (in this case 10) before the output value is retrieved. This procedure is employed in order to extract consistent output signals from networks with recurrent connections, which may fall into cyclic or chaotic attractors. Previously [32], it was found that allowing recurrent connections in morphology-generating CPPNs increased their evolvability. Voxel locations that exceed a predefined output threshold (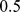 in this case) are considered to contain matter, while those that fall below this threshold are considered to be devoid of matter. All voxels lying on one of the extrema (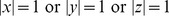) are given output value 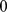 so that no matter-containing voxel abuts against the boundary of the grid, and therefore guarantees that the final triangular meshes have completely enclosed surfaces. Once the CPPN has been queried for every voxel location, the Marching Cubes algorithm [66] is employed to create triangular meshes from the underlying voxel data. Specifically, an enclosed triangular mesh is created for each connected voxel component which defines the exterior surface of a single physical shape. These triangular meshes are then sent to the physics simulator where they define the exterior surface of a solid object and are imbued with mass (see Fig. 1 for some examples). This is the first instance of physically simulating evolved, rigid body organisms composed of triangular meshes.

Since the purpose of this study is to investigate how different task environments affect the shapes of evolved morphologies, a number of simplifications are used in order to concentrate on the physical shapes of the evolved organisms and control for other factors that may influence their performance. From the multiple enclosed trimesh components that could be produced when querying a single CPPN, only one of these (the largest in terms of number of triangles) is used in the resulting organism. This single component is copied and reflected across the 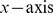. The resulting components (the original and its mirror image) are then spread apart by 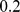 meters and a capsule of this length is placed between them such that it connects their two closest points. The two trimesh components each connect to this capsule by means of a hinge joint. These joints are constructed such that one rotates through the organism's coronal plane while the other rotates through its sagittal plane. Reflecting and copying a single component like this ensures that all organisms have the same mechanical degrees of freedom and ensures that the organisms are all bilaterally symmetric (which should facilitate locomotion) while at the same time it allows for a very large number of different morphologies due to the flexibility of the CPPN representation and trimesh model.

The two mechanical degrees of freedom of each organism are actuated by means of coupled oscillators. Each of the two oscillators is parameterized by several parameters: amplitude, period, and phase shift. These six parameters (three parameters apiece for each of the two joints) are directly encoded in the genome of the evolving organisms as floating point values so that the genome is in actuality a CPPN plus a six dimensional floating point array. These floating point values are recombined and mutated in the same manner as CPPN link weights with mutation magnitudes scaled by the range of values for that parameter. Additionally, crossover on these vectors is possible in all instances of sexual reproduction since every individual contains a vector of the same dimensionality. Values for these parameters are constrained to predefined ranges: amplitude, 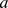 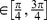 radians (so that the hinge rotates between 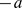 and *a* radians), period 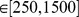 simulation time steps (or equivalently 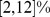 of the total evaluation time) and phase shift 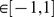 periods. Each parameter has a mutation probability of 0.1, which was chosen experimentally.

Encoding the control parameters in this fashion is done to keep the controllers as simple as possible so that fitness is primarily dictated by the physical form of the organisms, while at the same time allowing for diverse enough behavior so that the organisms can succeed in the different task environments.

### Selecting Desirable Organisms

The focus of this study is on how environment influences the evolution of morphological complexity in virtual organisms. Towards this aim a simple task is chosen which can be accomplished with more or less difficulty in a variety of environments. Specifically, as in previous work (e.g. [24], [25], [32], [67], [68]), the task investigated here is to maximize directed displacement in a fixed amount of time, across a range of terrains.

A candidate morphology (triangular mesh) and accompanying set of control parameters are sent to a physics simulator and allowed to act for a fixed number of simulation time steps. (In this work simulations are conducted in the Open Dynamics Engine (http://www.ode.org), a widely used open source, physically realistic simulation environment.) Since trimeshes can be arbitrarily shaped and, unlike spheres, may simultaneously contact the environment at several points, it is necessary to use a much smaller step size than has been used in previous work in order to get physically realistic behavior. Specifically, a step size of 0.001 s is used in this work. Because of this smaller step size a proportionally larger number of time steps are needed to achieve the same effective simulation length. Here organisms are evaluated for 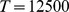 time steps.

#### Single-objective selection

After the organism has completed its time in the simulator its fitness is calculated. How exactly this fitness is calculated takes some care, because evolution often finds ways to “cheat” naïve fitness functions, especially when the task environment is difficult. For example, if fitness only considers the positions of the organism's center of mass, 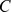, and takes fitness as 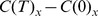 where 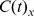 is the 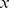-coordinate of the organism's center of mass at time 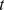 and 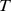 is the simulation length, then in environments where locomotion is difficult, evolution will tend to find solutions where 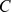 is initially raised far off the ground so that its displacement can be maximized by falling forward. This is a local optimum in this fitness landscape. Similarly, if one tries to eliminate this cheating by only considering the trailing point of the organism so that fitness is 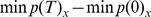 where 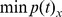 is the smallest 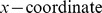 across all points on the organism at time 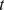, falling forward can still be an effective solution (and is still a local optimum) in difficult environments if morphologies are created which have posterior protrusions and thus make 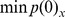 as small as possible.

In light of these considerations, the fitness function employed in all environments in this study is
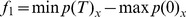
(1)where all coordinates are taken in terms of ODE units, which may be thought of as meters.

With this fitness function, falling forward will not be rewarded, because the maximum fitness that can be achieved by pivoting about a single point will be 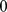, and so an organism must actually displace its whole body forward to be rewarded.

#### Multi-objective selection

In the initial set of experiments 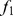 is the only fitness function employed, but in a second set of experiments 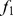 is maximized in conjunction with
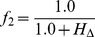
(2)through the use of multi-objective selection [51], [52]. 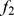 is strictly positive and is maximized for minimal values of 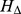.

By selecting for organisms that are both morphologically simple and are able to displace as far as possible in their given environment, it should be possible to evolve organisms that are no more complex than they need to be in order to succeed.

In order to evolve CPPNs using multi-objective selection it is necessary to modify CPPN-NEAT to use multiple fitness criteria. In lieu of the speciation and selection mechanisms employed by CPPN-NEAT, the widely used Non-dominated Sorting Genetic Algorithm-II (NSGA-II) [69] is used for selection. The two primary fitness functions to be maximized by NSGA-II are 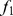, which selects for maximum fitness (see Eqn. 1) and 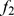, which selects for maximum morphological simplicity (see Eqn. 2). Additionally, preliminary experiments determined that including a genotypic diversity objective based on NEAT's compatibility metric consistently improved performance on both primary objectives, and so this additional objective is included in all reported experiments. It is thought that this term is useful because, like NEAT's speciation mechanism, it provides a means for solutions in different parts of the genotype space to evolve without competing directly with one another. (As detailed in [70] it is likely that performance could be improved even further by including a behavioral diversity metric, but it is not clear what an appropriate behavioral diversity metric is when evolving morphologies, while the NEAT compatibility metric is readily available.) Specifically, the genotypic diversity measure employed here (to be maximized) for each individual is calculated as the sum of the compatibility distances to its 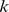 nearest neighbors. A value of 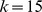 is employed in all experiments, because it was found to achieve the best performance during preliminary experimentation. However, when comparing the results of the multi-objective experiments (as is done in Figs. 6–8) the genotypic diversity objective is ignored, and only the Pareto front (see below) consisting of the individuals not dominated on the two primary objectives is considered for each trial.

Under this multi-objective framework each trial maintains a Pareto front (see Fig. 9) of non-dominated individuals representing various trade-offs between task competency and minimal complexity. An individual 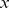 is said to dominate another individual 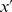 if 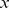 is not worse than 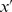 on any of the objectives and 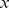 is strictly better than 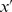 on at least one objective. The Pareto front contains all individuals 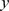 such that 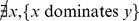.

**Figure 11:**
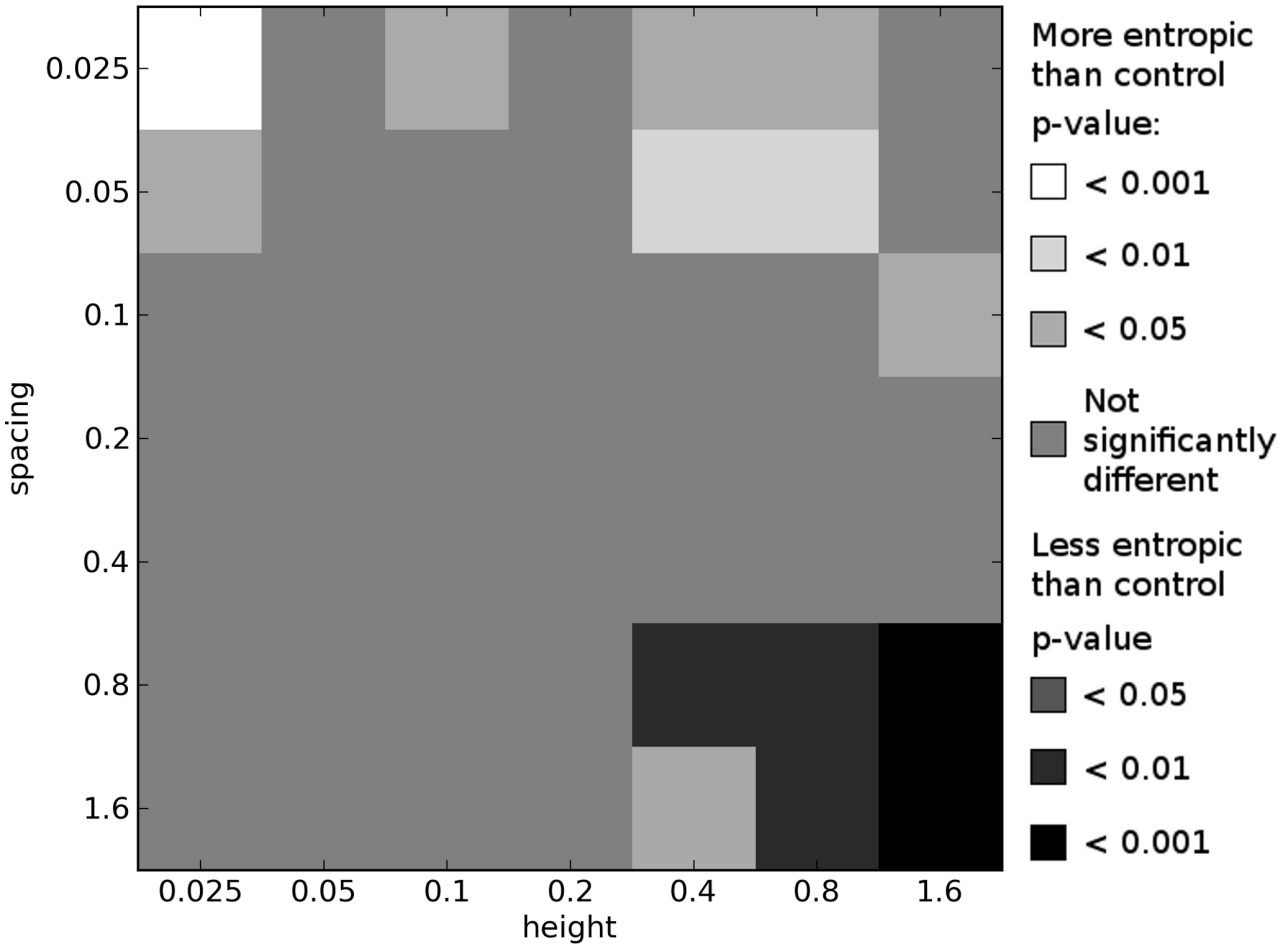
Differences in morphological complexity between icy and control environments: multi-objective best displacers. This plot compares the ways in which the complexity of morphologies from icy environments differ from the complexity of morphologies evolved in the control environment under multi-objective selection. This plot is created from the multi-objective results by comparing the 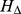 values of the best displacer in each trial's final Pareto front in each icy environment to the 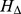 values of the best displacer in each trial's final Pareto front from the corresponding set of trials in the control environment. See Methods for details. All 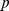-values calculated using the Mann-Whitney U test.

It is not entirely clear how these Pareto fronts should be compared. One possibility would be to consider the best displacers in each front (the far right point in Fig. 9). This is shown in Fig. 11. Here, some differences in the morphological complexities of organisms evolved in the icy environments versus flat ground can begin to be seen, but the effect is not wide spread, because the cost of complexity imposed on these individuals is weak. These individuals have little direct selection pressure favoring more simple bodies. Only when two equally good displacers are compared will the simpler one be favored. However, they are indirectly influenced in the direction of greater simplicity due to the way in which this objective is influencing the rest of the population. The fact that differences between the organisms evolved in icy and control environments begin to show up even under this weak cost of complexity further supports our conclusions.

Another possibility would be to find the knee point [71] on each Pareto front. The knee point is the point on the Pareto front which best captures a compromise between the objectives: the point at which a small gain in performance on one of the objectives would require a large drop in performance on the other objective. However, finding each knee point may capture drastically different levels of competencies on different fronts, and is not always well defined. In light of these considerations, we have adopted several methods of comparing the resulting Pareto fronts and demonstrate that the differences in complexities between morphologies evolved in the icy and control environments are similar with each method (thus demonstrating that the results are not an artifact of the particular method chosen).

### Calculating Morphological Complexity

In this section, the building blocks of computing the entropy of curvature 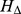 are presented. The reader is referred to [45], [46], [72], [73] for more in-depth discussions of their theoretical underpinnings.

Given a random variable 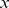 with a probability density function (PDF) 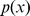, entropy 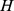 is defined as
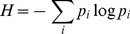
(3)where 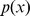 is discretized such that 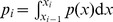 where the 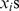 are specific values of 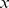.

Following [45], [46], the random variable 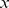 on which 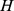 is calculated is an approximation of the Gaussian curvature of the points on the surface. (The Gaussian curvature 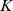 of a point is the product of the principal curvatures 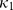 and 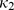 of that point [72].) Since the bodies here are built out of triangular meshes the points at which this curvature is non-zero are precisely the vertices of the triangular mesh. Specifically, for each vertex 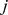 in a trimesh the angular deficit 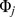 is calculated as
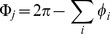
(4)where 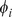 is the internal angle at 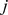 of each triangle 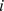 of which 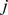 is a vertex. This angular deficit 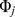 is directly proportional to the Gaussian curvature of that point [45], and so here we set 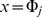 for calculating the entropy of curvature. (The relationship between angular deficit and Gaussian curvature can be derived through application of the Gauss-Bonnet theorem [72]; see [73] for more details.)

Following the calculation of 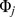 for every vertex, a PDF 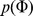 is estimated by placing the values of 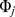 into discrete bins of uniform width (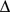) and counting the number of 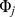 samples that fall into each bin. This results in a discrete set of probabilities 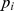, and Eqn. 3 can be used to arrive at an estimate of entropy that depends on the chosen 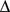, denoted here 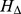. (see Text S1 for further details.)

### Code and Data

The source code used to run the experiments reported in this paper is publicly available at https://github.com/jauerb/CPPN_Trimesh

Additionally, the data files corresponding to the experiments reported in this paper have been made publicly available at http://dx.doi.org/10.6084/m9.figshare.858799

## Supporting Information

Figure S1 **Complexities and fitnesses over evolutionary time.** This plot depicts morphological complexity (*H*_Δ_) and fitness (displacement) over evolutionary time for all icy environments along with their corresponding set of trials from the control environment. For the icy environments morphological complexity is plotted in blue and displacement is plotted in red. For the corresponding trials in the control environment morphological complexity is plotted in black and displacement is plotted in green. Solid lines denote means (taken across all best of generation individuals from all trials in the set) and dotted lines denote one unit of standard error.
(TIF)

Figure S2 **How space filling the evolved morphologies are.** *Left*: Mean ratio between the volume of the evolved morphology's Axis Aligned Bounding Box (AABB) and the volume of the morphology itself for each of the experimental environments. The best organisms from all trials in the control environment have a mean of 3∶75 for this ratio, similar to the black squares in this plot. *Right*: Significance of the difference of this ratio in each experimental environment compared to the control environment. The ratio is significantly greater (morphologies are significantly less space filling) on average in the majority of experimental environments. There are no experimental environments in which this ratio is significantly smaller than that of the control. All p-values calculated using the Mann-Whitney U test.
(TIF)

Table S1 **Evolutionary Algorithm Parameters.**
(PDF)

Table S2 **Encoding Parameters.**
(PDF)

Table S3 **Compatibility Distance Parameters.**
(PDF)

Table S4 **Speciation Parameters.**
(PDF)

Table S5 **Multi-Objective Parameters.**
(PDF)

Table S6 **ODE Parameters.**
(PDF)

Text S1 **Supplementary materials.**
(PDF)

Video S1 **Video of an organism evolved in the at ground, control environment.** One of the best organisms evolved to locomote in the at ground environment. Notice that this organism has a very simple shape and is reminiscent of the blocky creatures evolved by Sims [19].
(MP4)

Video S2 **Video of an organism evolved in one of the icy environments.** One of the best organisms evolved to locomote in an environment with icy blocks 0.2 m tall spaced apart by 0.2 m. This organisms demonstrates how organisms evolve to be well adapted to their environment.
(MP4)

Video S3 **Video of an organism evolved in at ground and then placed in an icy environment.** This video depicts the organism shown in Video S1 in the icy environment shown in Fig. S2. Notice that this organism does not have an appropriate morphology for this environment and is unable to successfully locomote. This organism continues to be unable to locomote even if its control parameters are re-evolved in this environment.
(MP4)

Video S4 **Video of another one of the best organisms evolved in the at ground, control environment.** This is the same organism depicted in the top left of Fig. 1.
(MP4)

Video S5 **Video of one of the best organisms evolved in an icy environment with blocks 0.8 m tall and spaced apart by 0.025 m.** This is the same organism depicted in the top right of Fig. 1.
(MP4)

Video S6 **Video of one of the best organisms evolved in an icy environment with blocks 0.4 m tall and spaced apart by 0.2 m.** This is the same organism depicted in the bottom left of Fig. 1.
(MP4)

Video S7 **Video of one of the best organisms evolved in an icy environment with blocks 0.2 m tall and spaced apart by 0.8 m.** This is the same organism depicted in the bottom right of Fig. 1.
(MP4)

Video S8 **Video of one of the best organisms evolved in an icy environment with blocks 0.05 m tall and spaced apart by 1.6 m.**
(MP4)

Video S9 **Video of one of the best organisms evolved in an icy environment with blocks 0.2 m tall and spaced apart by 0.1 m.**
(MP4)

Video S10 **Video of one of the best organisms evolved in an icy environment with blocks 0.2 m tall and spaced apart by 0.4 m.**
(MP4)

Video S11 **Video of one of the best organisms evolved in an icy environment with blocks 0.4 m tall and spaced apart by 0.05 m.**
(MP4)

Video S12 **Video of one of the best organisms evolved in an icy environment with blocks 0.4 m tall and spaced apart by 0.1 m.**
(MP4)

Video S13 **Video of one of the best organisms evolved in an icy environment with blocks 0.4 m tall and spaced apart by 0.4 m.**
(MP4)

Video S14 **Video of one of the best organisms evolved in an icy environment with blocks 0.8 m tall and spaced apart by 0.4 m.**
(MP4)

Video S15 **Video of one of the best organisms evolved in an icy environment with blocks 1.6 m tall and spaced apart by 0.1 m.**
(MP4)
