# Supplementary figures and images for: Environmental Influence on the Evolution of Morphological Complexity in Machines
Source: PLoS Comput Biol. 2014 Jan 2;10(1):e1003399. doi: 10.1371/journal.pcbi.1003399 (PMC3879106; doi:10.1371/journal.pcbi.1003399)

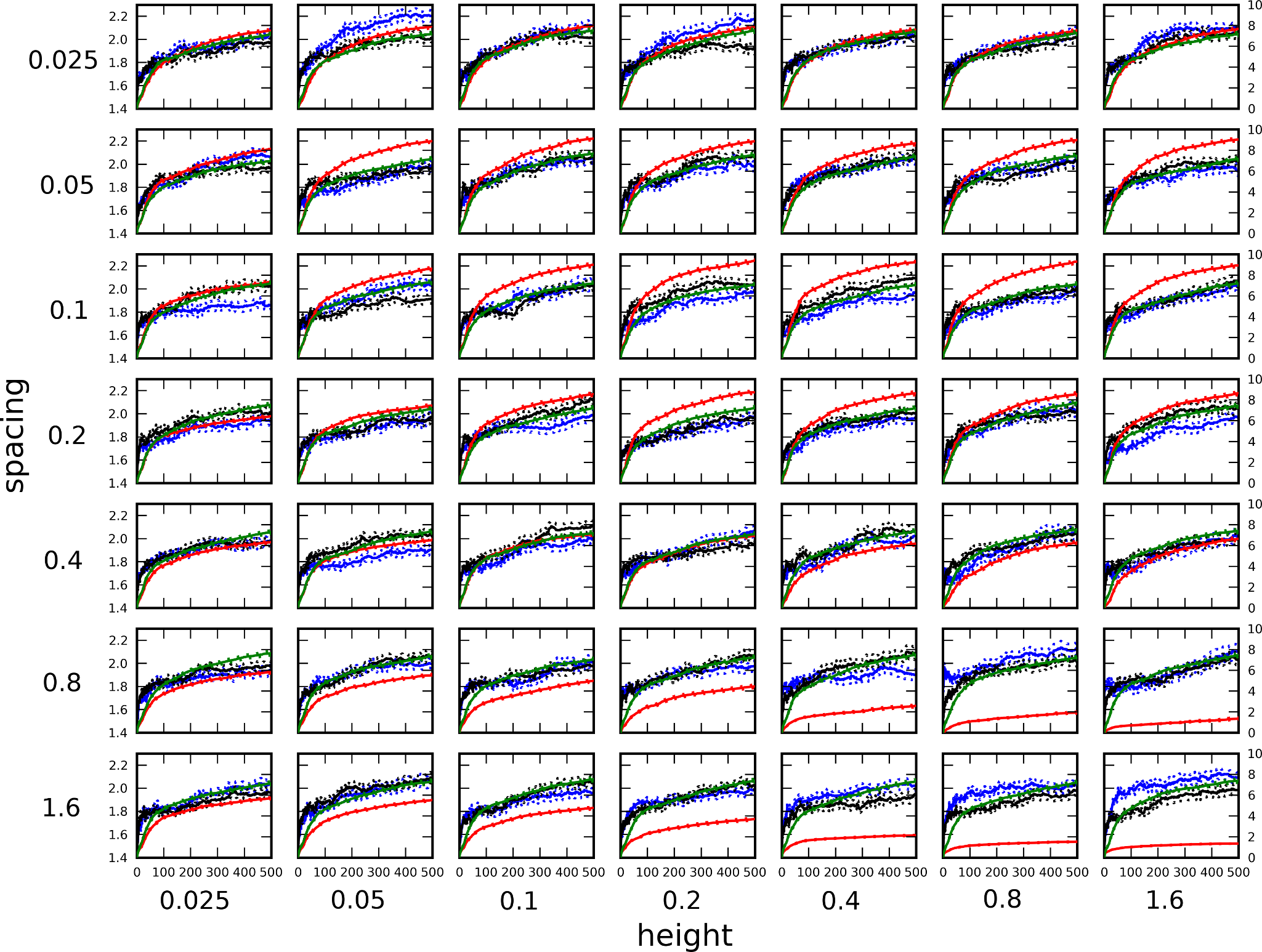

Supplement: Figure S1 — Complexities and fitnesses over evolutionary time. This plot depicts morphological complexity (HΔ) and fitness (displacement) over evolutionary time for all icy environments along with their corresponding set of trials from the control environment. For the icy environments morphological complexity is plotted in blue and displacement is plotted in red. For the corresponding trials in the control environment morphological complexity is plotted in black and displacement is plotted in green. Solid lines denote means (taken across all best of generation individuals from all trials in the set) and dotted lines denote one unit of standard error. (TIF) [file pcbi.1003399.s001.tif]

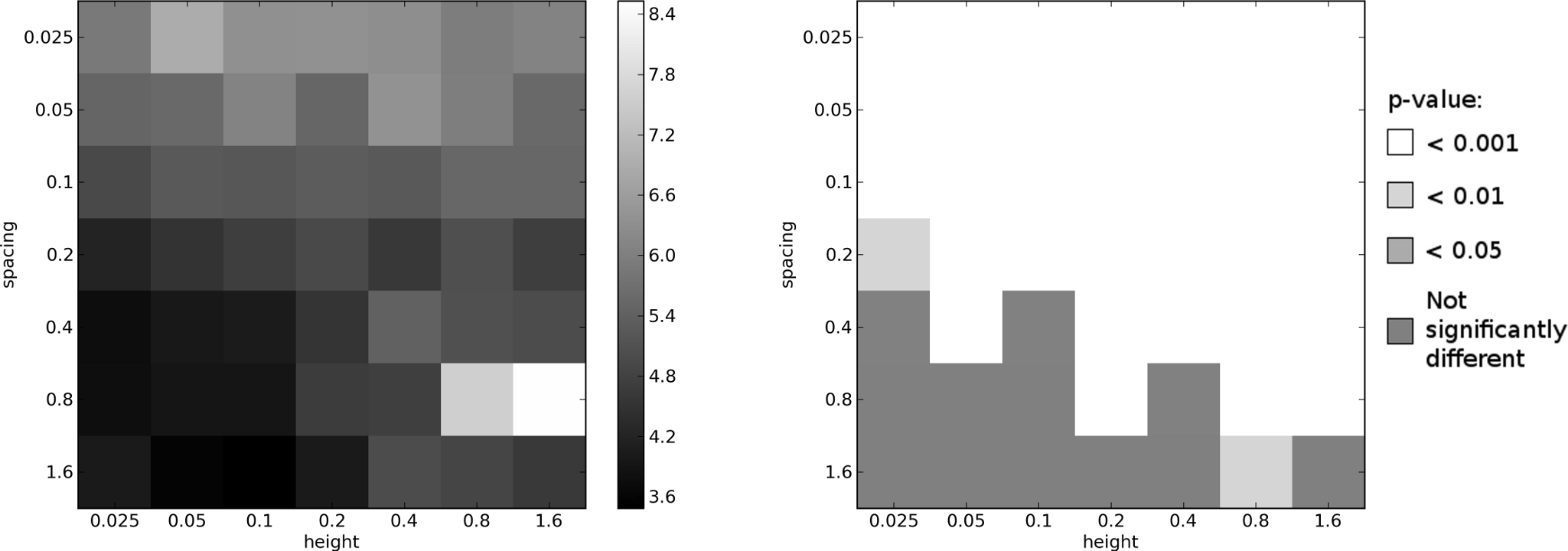

Supplement: Figure S2 — How space filling the evolved morphologies are. Left: Mean ratio between the volume of the evolved morphology's Axis Aligned Bounding Box (AABB) and the volume of the morphology itself for each of the experimental environments. The best organisms from all trials in the control environment have a mean of 3∶75 for this ratio, similar to the black squares in this plot. Right: Significance of the difference of this ratio in each experimental environment compared to the control environment. The ratio is significantly greater (morphologies are significantly less space filling) on average in the majority of experimental environments. There are no experimental environments in which this ratio is significantly smaller than that of the control. All p-values calculated using the Mann-Whitney U test. (TIF) [file pcbi.1003399.s002.tif]
