# Supplementary material for: Environmental Influence on the Evolution of Morphological Complexity in Machines
Source: PLoS Comput Biol. 2014 Jan 2;10(1):e1003399. doi: 10.1371/journal.pcbi.1003399 (PMC3879106; doi:10.1371/journal.pcbi.1003399)
Supplement: Table S1 — Evolutionary Algorithm Parameters. (PDF) [file pcbi.1003399.s003.pdf]

**Parameter Name**	**Value**
Population Size	150
Max Generations	500
Mutate Add Node Probability	0.03
Mutate Add Link Probability	0.05
Mutate Demolish Link Probability	0.00
Mutate Link Weights Probability	0.8
Mutate Only Probability	0.25
Mutate Link Probability	0.1
Allow Add Node To Recurrent Connection	No
Mutation Power	2.5
Adult Link Age	18.0
Allow Recurrent Connections	Yes
Allow Self Recurrent Connections	No
Force Copy Generation Champion	Yes
Link Gene Minimum Weight For Phenotype	0.0
