# Supplementary material for: Environmental Influence on the Evolution of Morphological Complexity in Machines
Source: PLoS Comput Biol. 2014 Jan 2;10(1):e1003399. doi: 10.1371/journal.pcbi.1003399 (PMC3879106; doi:10.1371/journal.pcbi.1003399)
Supplement: Table S2 — Encoding Parameters. (PDF) [file pcbi.1003399.s004.pdf]

**Parameter Name**	**Value**
Number of CPPN Update Iterations	10
CPPN Activation functions	Signed cosine, Gaussian, and sigmoid
Matter Threshold	0.5
Amplitude Range	$\left[\frac{\pi}{4}, \frac{3\pi}{4}\right]$
Period Range	$[250, 1500]$
Phase Shift Range	$[-1, 1]$
