# Supplementary material for: Environmental Influence on the Evolution of Morphological Complexity in Machines
Source: PLoS Comput Biol. 2014 Jan 2;10(1):e1003399. doi: 10.1371/journal.pcbi.1003399 (PMC3879106; doi:10.1371/journal.pcbi.1003399)
Supplement: Table S4 — Speciation Parameters. (PDF) [file pcbi.1003399.s006.pdf]

**Parameter Name**	**Value**
Compatibility Threshold	6.0
Compatibility Modifier	0.3
Species Size Target	8
Dropoff Age	15
Age Significance	8.0
Survival Threshold	0.2
Smallest Species Size With Elitism	5
Mutate Species Champion Probability	0.0
