# Supplementary material for: Environmental Influence on the Evolution of Morphological Complexity in Machines
Source: PLoS Comput Biol. 2014 Jan 2;10(1):e1003399. doi: 10.1371/journal.pcbi.1003399 (PMC3879106; doi:10.1371/journal.pcbi.1003399)
Supplement: Table S5 — Multi-Objective Parameters. (PDF) [file pcbi.1003399.s007.pdf]

**Parameter Name**	**Value**
Include Genotypic Diversity Objective	Yes
Genotypic Diversity k	15
