# Supplementary material for: Environmental Influence on the Evolution of Morphological Complexity in Machines
Source: PLoS Comput Biol. 2014 Jan 2;10(1):e1003399. doi: 10.1371/journal.pcbi.1003399 (PMC3879106; doi:10.1371/journal.pcbi.1003399)
Supplement: Table S6 — ODE Parameters. (PDF) [file pcbi.1003399.s008.pdf]

**Parameter Name**	**Value**
Step Size	0.001s
Evaluation Length	12500 time steps
Contact Surface μ (ice)	0
Contact Surface μ (ground)	*dInfinity*
Contact Surface Slip1	0.01
Contact Surface Slip2	0.01
Contact Surface Soft ERP	0.96
Contact Surface Soft CMF	0.01
Contact Max Correcting Velocity	0.01
Contact Surface Layer	0.001
Motor FMax	0.5
Motor K_p	3
Motor K_d	0
Motor K_i	0
Gravity Vector	$(0, 0, -9.8)$
Linear Damping	0.005
Angular Damping	0.005
Trimesh Density	0.1
Capsule Density	5.0
