# Supplementary material for: Environmental Influence on the Evolution of Morphological Complexity in Machines
Source: PLoS Comput Biol. 2014 Jan 2;10(1):e1003399. doi: 10.1371/journal.pcbi.1003399 (PMC3879106; doi:10.1371/journal.pcbi.1003399)
Supplement: Text S1 — Supplementary materials. (PDF) [file pcbi.1003399.s009.pdf]

Text S1

Axis Aligned Bounding Box

One simple way to study how environments select for more or less complex morphologies is to consider how space filling the evolved morphologies are. This can be done by computing the ratio of the volume of a morphology's Axis Aligned Bounding Box (AABB) to the volume of that morphology itself. (For simplicity all morphological measures are computed on the single enclosed trimesh object that is produced by Marching Cubes for a CPPN, i.e. the reflected copy of this trimesh and the connecting capsule are not considered.)

Fig. S2 shows the mean values of this ratio, once again taken across the 100 best of trial individuals from each experimental environment. Also plotted is how significantly different this ratio is, on average, in each experimental environment when compared to the best of trial individuals from the corresponding set of trials in the control environment. In the majority of experimental environments this ratio is significantly greater from that of the organisms in the control experiment. This demonstrates that these environments do in fact influence the morphologies of the organisms which evolve inside them in quantifiable ways: they are more space filling than those evolved in the control environment for a large portion of the parameter space. Additionally, Fig. S2 (left) shows how (at least) one aspect of how morphology gradually changes as one moves through this environmental parameter space: the evolved morphologies become less space filling as height increases and spacing decreases. This lends support to the chosen parameterization being a good one for the purpose of studying how the morphologies of organisms are affected by the environment in which they evolve.

Reproduction Depth-Controlled Shadow Models

As described in the text, the naïve shadow model (random selection) does not provide a good control case for investigating the changes in morphological complexity over time because at a given generation individuals in the random selection experiments will be the end product of many more reproduction (mutation and crossover) events than the corresponding individuals evolving for displacement. This is because the majority of mutations are detrimental for a real fitness objective, and so it is likely that newly

introduced individuals will be discarded in the displacement experiments, while under random selection they are just as likely to reproduce as any other individual. Therefore, these individuals will have had many more opportunities to add to the complexity of their genomes and hence the complexity of their morphologies. In order to create a neutral shadow model that does not suffer from this bias, alternative reproduction depth controlled shadow models are created.

For each independent trial of CPPN-NEAT for which shadow models will be run a record of every reproduction event is created as follows: When going from generation t to generation $t+1$ there are survivors (S), mutants (M), and individuals resulting from crossover (C). For each instance of each of these classes the number of mutation and crossover events in the ancestral line leading to that individual is recorded. So for an individual $s \in S$, whose evolutionary history contains i mutations and j crossovers, an entry (i, j) is added to the list of survivors at generation t and s maintains that it had i mutations and j crossovers. Similarly if individual m produces mutated offspring m' (either by adding additional nodes or links, or by altering existing genetic material), and in the evolutionary history of m there were i mutations and j crossovers, an entry (i, j) is added to the list of mutants at generation t and m' then gets recorded as having $i' = i + 1$ mutations and $j' = j$ crossovers. Finally if c_1 and c_2 with histories (i_1, j_1) and (i_2, j_2) cross to form c', an entry $((i_1, j_1), (i_2, j_2))$ is added to the list of crossovers at generation t and c' gets recorded as having $i' = \max(i_1, i_2)$ mutations and $j' = \max(j_1, j_2) + 1$ crossovers.

For each trial of CPPN-NEAT, a reproduction depth controlled shadow model is run whereby at each generation individuals that match the reproduction profiles of the trial being shadowed are randomly selected. For example if at generation t in the real trial there were k survivors with evolutionary histories $(i_1, j_1), (i_2, j_2), ...(i_k, j_k)$ then in the shadow model there will also be k survivors, the first of which is randomly selected from all individuals in the current population which are the result of i_1 mutations and j_1 crossovers, the second of which is randomly selected from all individuals in the current population which are the result of (i_2, j_2) reproduction events, and so on. The same is done for the mutation and the crossover events. Running a shadow model in this fashion guarantees that each real trial will have a shadow where selection is random, but at each generation there are individuals with matching numbers of mutation and crossover events.

There are a few additional caveats here. First, in order to compute morphological complexities, valid morphologies are needed: the CPPN must output values above the threshold at some subset of the queried locations. So, if there are individuals matching the different reproduction events that also

produce valid morphologies, those are given preference over those individuals that do not produce valid morphologies (similarly to what is done for completely random selection). Second, while there will always be individuals in the population matching the appropriate histories (since they are being followed from the outset) the speciation in CPPN-NEAT is dynamic and so there might not be individuals who match the crossover profiles and are in the same species. Crossing over between species is not something that would ever happen in an actual trial of CPPN-NEAT so this may be problematic. To compensate for this, two different shadow models are run for each actual trial of CPPN-NEAT.

In shadow model a, for each crossover event the reproduction profiles are matched exactly with preference given to matching individuals within the same species, but if there are no matching individuals that are also in the same species crosses between individuls from different species is used as a fall back. In shadow model b inter-species crosses are never allowed (so its behavior is more in line with CPPN-NEAT), but this means it may be necessary to allow some flexibility in matching the reproduction profiles. Here, the reproduction profiles of crossovers are matched exactly if possible (i.e. appropriate individuals within the same species exist), but otherwise progressively larger deviations from the reproduction profiles are allowed until an appropriate intra-species crossover is found. These deviations can cascade though and so it is necessary to follow the same procedure of allowing progressively larger deviations in reproduction profiles for survivors and mutations as well. Shadow model b eliminates any bias that might be introduced from allowing inter-species crosses, but may introduce its own biases by not matching the reproduction profiles exactly. However, both models have similar complexity curves (see Fig. 8) indicating that neither bias has a large effect, and that this shadow procedure is robust to whichever alternative is employed.

H_Δ Considerations

As mentioned in the main text, the result of the morphological complexity metric H_Δ depends on the choice of Δ. If Δ is too large the majority of samples will fall into the same bin and all information is lost. If Δ is too small then the majority of samples will fall into independent bins and H_Δ reduces to a function of the number of vertices n. In general there is no optimum Δ, and since the trimesh morphologies considered here have much fewer vertices than those of [45] a correspondingly larger bin width must be used. In all calculations presented in this work a bin width $\Delta = \frac{\pi}{10}$ is used, chosen as a reasonable value by visually inspecting histograms of varying bin widths.
